# Plant-level intensity of energy and CO_2_ emissions for Portland cement in Guizhou of Southwest China 2019–2022

**DOI:** 10.1038/s41597-024-03621-5

**Published:** 2024-07-11

**Authors:** Wenhao Wang, Minghua Ye, Yanfang Shi, Dongchen Xiao

**Affiliations:** 1 School of Materials & Metallurgy, Guizhou University, Guiyang, 550025 China; 2 Guizhou Qianlonghui Technological Company, Guiyang, 550009 China; 3 Western Development Service Center of Qiannan City of Guizhou, Qiannan, 558000 China

**Keywords:** Environmental impact, Energy efficiency

## Abstract

Low-carbon development of ordinary Portland cement industry is of great significance to China’s target “to peak carbon dioxide emissions before 2030 and to achieve carbon neutrality before 2060”. Neglecting the regional heterogeneity in China, few studies emphasized the status and developments of energy intensity and CO_2_ emissions of ordinary Portland cement industry in Guizhou of Southwest China. To bridge this knowledge gap, we present an annual intensity dataset of energy and CO_2_ emissions at plant-level for Guizhou’s ordinary Portland cement industry, which involves the details of clinker rotary kilns, yearly production of clinker and cement products, fuel consumption and electricity consumption, total CO_2_ emission of cement products, energy intensity indicators of clinker and cement products, utilization ratio of solid-waste in clinker and cement production, and CO_2_ emission factors of cement products. It is an important supplement and to existing energy intensity and CO_2_ emissions estimates at plant-level and provincial official emissions inventories that converges all regions of China.

## Background & Summary

Ordinary Portland cement (OPC cement) is a crucial material for civil and building engineering and is widely used in making concrete, mortar, and other products^1^. More than 98% of the OPC cement plants in China introduced the new suspension preheating (NSP) process in the last ten years. In the NSP process, the raw meals proportioned by milled limestone and siliceous ores are heated in a rotary kiln to first thermal dissociate calcium carbonate in limestone to calcium oxide, and then the calcium oxide reacts with silicon ores to form alite and belite for the clinker, while the temperature in the rotary kiln is up to 1400 °C and usually fired with fossil fuels^2^. The intermediate product clinker would be further milled with gypsum and other mineral admixtures, accounting for about ~25.0%, in the grinding mills to yield the OPC cement^3^.

The OPC cement industry is one of the most energy-intensive industries and generates a large amount of CO_2_ emissions, responding to about 7.0% of China’s total energy consumption and about 15.0% of total CO_2_ emissions without significant variation annually^4–6^. More specially, about 0.4962~1.0015 ton^7^, of CO_2_ emissions that both for decarboxylation-derived and energy-derived CO_2_ emissions^8^, are emitted per ton of the OPC cement produced with differences in materials of clinker and cement products, type of chosen clinker kiln, and burned fuels of kilns^9^. Thus, increasing attention should be paid to the national OPC cement-related CO_2_ emissions, while the national production of OPC cement is approximately 21.3 billion tons in 2022 and share 51.2% of global OPC cement production^10^, and the realization of low-carbon development of the OPC cement industry will be of great significance to China’s target “to peak carbon dioxide emissions before 2030 and to achieve carbon neutrality before 2060”^11^.

Past studies estimate that there are three major approaches to mitigating CO_2_ emissions in the OPC cement industry to promote green transition and sustainable development^2^. Firstly, energy efficiency measures, such as new large-scale dry kilns and waste heat power generation technology to recover energy and waste heat^12^, are not only the basic strategy to improve energy intensity to enhance sustainability but also to reduce CO_2_ emissions. Secondly, alternative fuels, such as municipal domestic waste, biomass, and coal gangue, are used in the rotary kiln^13,14^, and alternative materials, such as fly ash, phosphogypsum, desulfurization gypsum, construction & demolition waste, and mineral slags & coal cinder^15,16^, are substituted to reduce dependency on clinker to upgrade energy intensity of cement and reduce CO_2_ emissions effectively^17,18^ that not only energy-derived but also decarboxylation-derived. Lastly, the planned numerous pilots and larger-scale demonstrations for carbon capture and storage (CCS) have significant potentiality^19,20^.

**Fig. 1:**
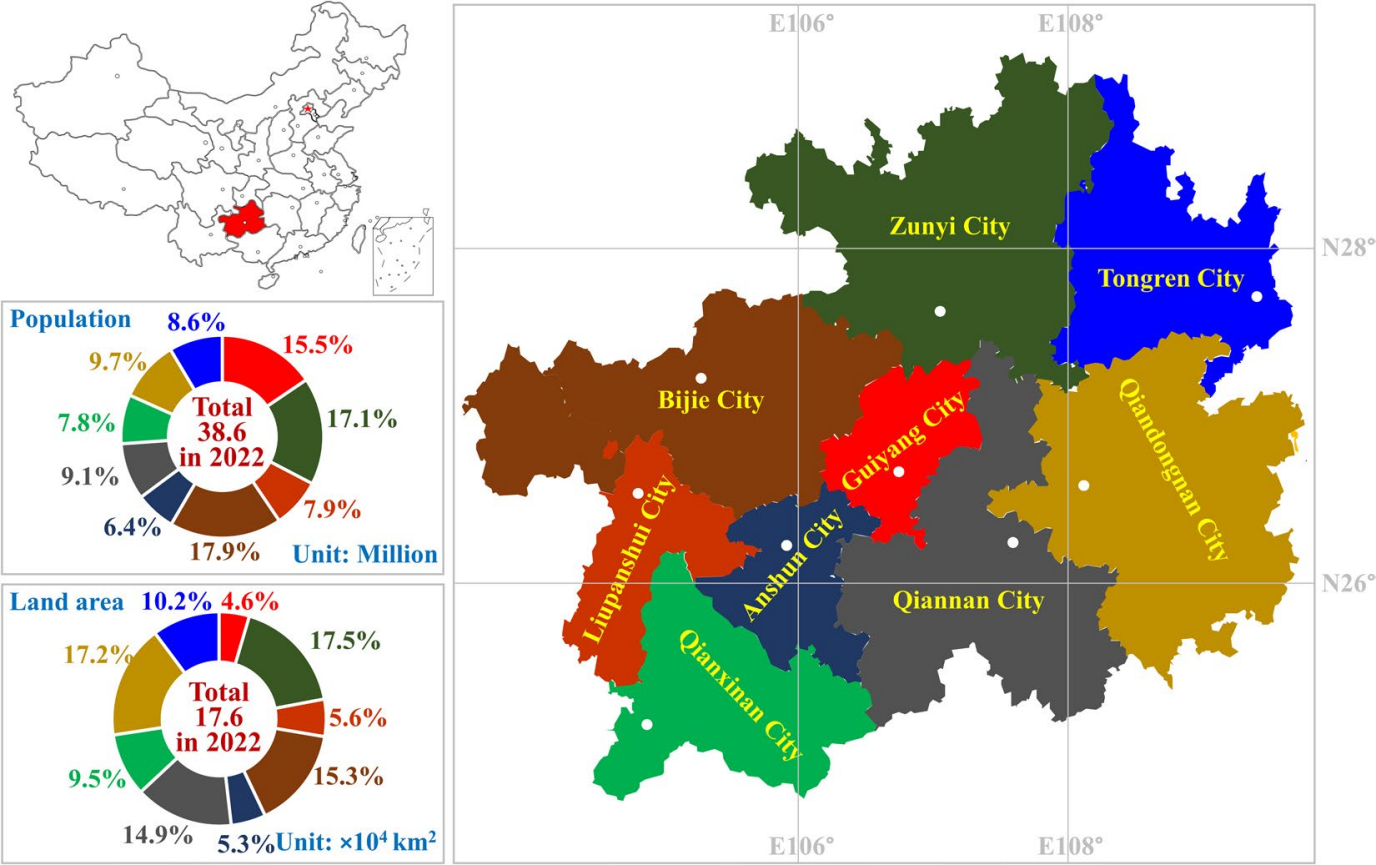
Location and administrative area of Guizhou province in Southwest China.

As a typical less-developed mountainous region and the important ecological barrier in the upper reaches of the Yangtze River and the Pearl River, Guizhou province, located in Southwest China (Fig. 1), faces common structural environmental problems and special problems of serious lagging of infrastructure for ecological environment protection. More specifically, Guizhou not only needs to speed up its development and consolidate its achievements in poverty alleviation but also faces the challenges of dwindling resources and a degrading environment. Guizhou has high levels of coal, phosphorite, bauxite, and manganese in its soil, and a large amount of fly ash, red mud, and phosphogypsum accumulated in the related industrial production process share about 2.3~2.8% of the national general industrial solid-wastes, which results in the serious environmental pollution in their substantial discharge and stockpiling. Guizhou has also been responsible for 4.0~5.0% of China’s total OPC cement production in recent years^21^, which could utilize a considerable amount of that solid-waste to reduce the demands for fuels and raw materials. The benefit of utilization of solid-waste from its OPC cement plants has not yet been systematically assessed, owing to limited data^22,23^.

To support the development of an effective carbon peaking policy implemented by provincial and county authorities and realize the energy and environmental benefits, it is necessary to compile an accurate dataset of the CO_2_ emissions from China’s OPC cement production that attracts worldwide attention. However, most studies emphasized the developments and status of that developed provinces or cities over backward Guizhou and Yunnan, neglecting the regional heterogeneity in China^24–26^, which cannot support the precise implementation of their high-quality development targets^26^. Thus, to the bridge knowledge gap, not only for the discrepancies and uncertainties, in the data for the OPC cement of the developing regions at the provincial or plant-level, we present an annual intensity dataset of energy and CO_2_ emissions at plant-level for the OPC cement production in Guizhou of Southwest China from 2019 to 2022. This dataset involves the details of clinker rotary kilns, yearly production of the clinker and OPC cement products, fuel consumption and electricity consumption, total CO_2_ emissions of the OPC cement products, energy intensity indicators of the clinker and OPC cement products, utilization ratio of the solid-waste in clinker and cement production, and CO_2_ emissions factors of the OPC cement products. It is also an important supplement and to existing energy intensity and CO_2_ emissions estimates at plant-level and provincial official emissions inventories that converges all regions of China.

## Methods

### Boundary definition and overview of OPC cement plants in Guizhou

The Ministry of Industry and Information Technology of China (MIIT) has achieved full coverage of thousands of the OPC cement plants through the industrial energy conservation inspections since 2016 (https://www.miit.gov.cn/jgsj/jns/wjfb/art/2020/art_9db88c4f2bfe45d1adc651130bf3c541.html). From 2017, most municipal supervisory authorities of nine cities in Guizhou independently conducted the work of the industrial energy conservation inspections, including but not limited to cement plants, iron-steel plants, non-ferrous metallurgy plants, and thermal power plants. Although national and provincial work manuals for the industrial energy conservation inspections have been developed and established (https://www.miit.gov.cn/n1146285/n1146352/n3054355/n3057542/n3057545/c5230497/part/5230510.pdf), there are still cognitive differences for the same industry in the monitoring groups organized in different cities. Since 2019, the Department of Industry and Information Technology of Guizhou province (DIIT of Guizhou) has organized a professional monitoring group for each industry to ensure the integrity and authority in the work of the industrial energy conservation inspections. Thus, the temporal boundary starts from 2019 in this data descriptor.

**Fig. 2:**
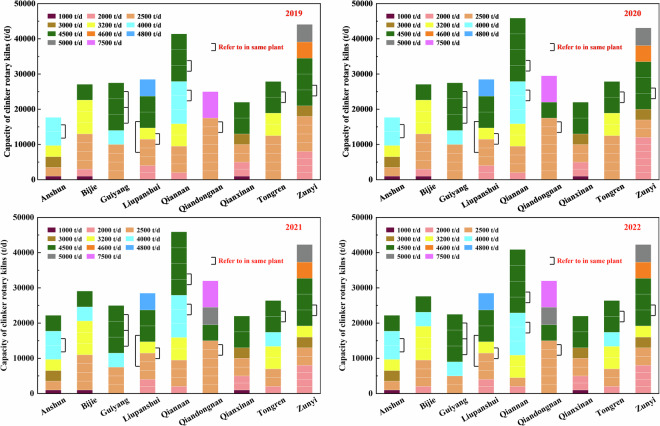
Details of city-level clinker rotary kilns for OPC cement plants in Guizhou from 2019 to 2022.

The spatial boundary of this dataset covers the whole territory of Guizhou province, including Anshun City, Bijie City, Guiyang City, Liupanshui City, Qiannan City, Qiandongnan City, Qianxinan City, Tongren City, and Zunyi City. There are 74, 76, 75, and 75 OPC cement plants covering nearly 84, 86, 85, and 85 clinker rotary kilns all produced by the NSP process distributed steadily in the above nine regions in Guizhou province from 2019, respectively. Several plants have two or three clinker rotary kilns. There is a significant spatial discrepancy in capacity and quantity of clinker rotary kilns. Nearly half of the total capacity of clinker rotary kilns is still provided by outdated equipment whose production capacity less than 4000 t/d in clinker calcination. Figure 2 shows the city-level distributions of different clinker rotary kilns for the OPC cement plants from 2019.

### Data collection and management

**Fig. 3:**
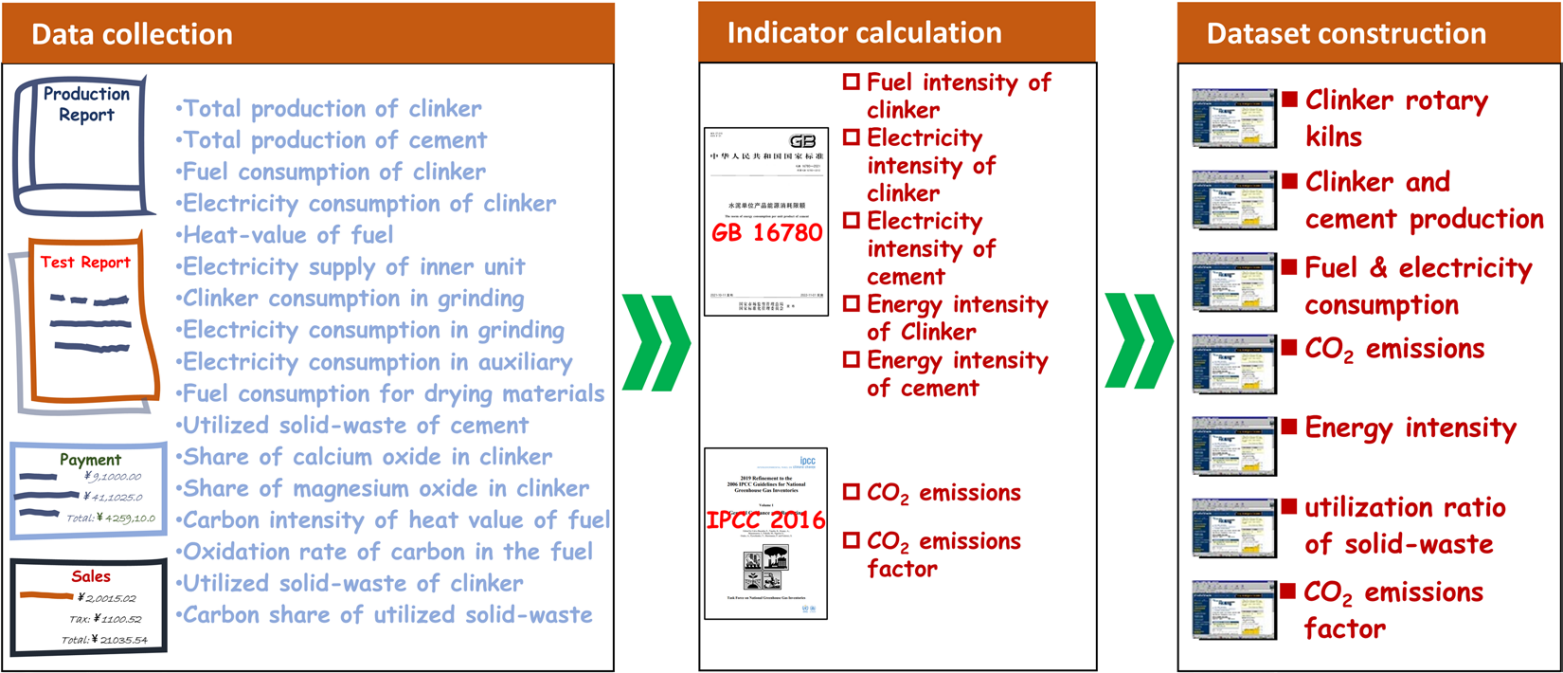
Flowchart of entire processing work of this dataset.

The primary yearly plant-level data from 2019 are mainly investigated and obtained from the special supervision for tiered pricing of electricity of cement plant, regarded as an indispensable part of the national industrial energy conservation inspections. The collected primary data includes the various fuel consumption, heat-value and carbon content of the fuel consumption, total production of clinker and OPC cement, electricity consumption, electricity supply of waste heat recovery unit, shares of calcium oxide and magnesium oxide in the clinker product, and details of utilized solid-waste. The energy intensity, CO_2_ emissions or emissions factors, and utilization ratio of solid-waste, regarded as secondary data, are generally calculated from the primary data followed by the national standard of *The Norm of Energy Consumption per Unit Product of Cement* at https://std.samr.gov.cn/gb/search/gbDetailed?id=E116673EA6DAA3B7E05397BE0A0AC6BF, and *2006 IPCC Guidelines for National Greenhouse Gas Inventories* at https://www.ipcc-nggip.iges.or.jp/public/2019rf/index.html. The entire processing work of the present dataset is described in Fig. 3.

The relative uncertainties of those primary data are very low, considered to be or less than 1.0%, while those data were collected from openly available data of the OPC cement plants. The error propagation methods^27,28^, an alternative faster and more reliable than the Monte Carlo method within the same indicators, are used to calculate the relative uncertainties of the secondary data.

### Energy intensity and CO_2_ emissions for OPC cement product

According to the national standard of *The Norm of Energy Consumption per Unit Product of Cement*, the assessment the energy intensity indicators of the OPC cement products is estimated in Table 1.

**Table 1:** Assessment of energy intensity indicators of OPC cement product.

Data	Sources or calculation	Relative uncertainty
Fuel consumption of clinker: *m*_*i*_	Filed investigation	1.0%
Heat-value of fuel: *Q*_*i*_	Filed investigation	0.5%
Heat-value of coal equivalent: *Q*_*ce*_	29307.6 kJ/kg	
Total production of clinker: *P*_*cl*_	Filed investigation	1.0%
Electricity consumption of clinker: *E*_*cl*_	Filed investigation	0.3%
Electricity supply of waste heat recovery unit: *E*_*re*_	Filed investigation	0.3%
Fuel intensity of clinker: *EF*_*cl*_	*EF*_*cl*_ = ∑(*m*_*i*_ × *Q*_*i*_)/(*Q*_*ce*_ × *P*_*cl*_) − 0.1229 × *E*_*re*_*/P*_*cl*_	2.1%
Electricity intensity of clinker: *EE*_*cl*_	*EE*_*cl*_ = *E*_*cl*_/*P*_*cl*_	1.1%
Energy intensity of clinker: *EI*_*cl*_	*EI*_*cl*_ = *EF*_*cl*_ + 0.1229 × *EE*_*cl*_	2.3%
Clinker consumption in grinding process: *P*_*cl-in*_	Filed investigation	1.0%
Total production of cement: *P*_*ce*_	Filed investigation	1.0%
Clinker to cement: *η*	*η* = *P*_*cl-in*_*/P*_*ce*_	1.4%
Electricity consumption in grinding process: *E*_*grind*_	Filed investigation	0.3%
Electricity consumption in auxiliary process: *E*_*au*_	Filed investigation	0.3%
Fuel consumption for drying raw materials of cement: *F*_*ce*_	Filed investigation	1.1%
Utilized solid-waste of cement: *W*_*ce*_	Filed investigation	1.0%
Electricity consumption of cement: *E*_*ce*_	*E*_*ce*_ = *E*_*grind*_ + *E*_*au*_* + P*_*cl-in*_ × *EE*_*cl*_	1.5%
Electricity intensity of cement: *EE*_*ce*_	*EE*_*ce*_ = *E*_*ce*_*/P*_*ce*_	1.8%
Energy intensity of cement: *EI*_*ce*_	*EI*_*cl*_ = *η* × *EI*_*cl*_ + *F*_*ce*_/*P*_*ce*_ + 0.1229 × *EE*_*ce*_	3.6%

The CO_2_ emissions in the OPC cement plants mainly come from the decarboxylation of calcium carbonate and magnesium carbonate in the raw materials for cement products, fuel combustion, and external electricity consumption. According to *2006 IPCC Guidelines for National Greenhouse Gas Inventories*, the assessment of CO_2_ emissions of the OPC cement products is shown in Table 2.

**Table 2:** Assessment of CO_2_ emissions of OPC cement products.

Data	Sources or calculation	Relative uncertainty
Share of calcium oxide in clinker product: *C*_CaO_	Filed investigation	1.0%
Share of magnesium oxide in clinker product: *C*_MgO_	Filed investigation	1.0%
Decarboxylation-derived CO_2_ emission: *C*_*de*_	*C*_*de*_ = (*C*_CaO_ × 44.0/56.1 + *C*_MgO_ × 44.0/40.3) × *P*_*cl*_	1.7%
Carbon intensity of heat value of fuel: *CC*_*i*_	Filed investigation	1.0%
Oxidation rate of carbon element in the fuel: *OF*_*i*_	Filed investigation	1.0%
Fuel-derived CO_2_ emissions: *C*_*fu*_	*C*_*fu*_ = ∑(*m*_*i*_ × *Q*_*i*_ × *CC*_*i*_ × *OF*_*i*_ × 44.0/12.0)	1.8%
Utilized solid-waste of clinker: *W*_*cl*_	Filed investigation	1.0%
Carbon share of utilized solid-waste in kiln: *CC*_*sw*_	Filed investigation	1.0%
Solid-waste-derived CO_2_ emission: *C*_*sw*_	*C*_*sw*_* = *∑(*W*_*cl*_ × *CC*_*sw*_ × 44.0/12.0)	1.4%
External electricity consumption: *E*_*net*_	*E*_*net*_ = *E*_*grind*_ + *E*_*au*_* + E*_*cl*_*-E*_*re*_	0.6%
CO_2_ emissions factor of electricity: *CF*_*e*_	Calculated from *Guizhou Statistical Yearbook*	1.7%
Electricity-derived CO_2_ emissions: *C*_*el*_	*C*_*el*_ = *E*_*net*_ × *CF*_*e*_	1.8%
Total CO_2_ emissions of cement: *C*_*ce*_	*C*_*ce*_ = *C*_*de*_ + *C*_*fu*_ + *C*_*el*_ + *C*_*sw*_	3.4%
CO_2_ emissions factor of cement: *CI*_*ce*_	*CI*_*ce*_ = *C*_*ce*_*/P*_*ce*_	3.5%
Utilization ratio of solid-waste in clinker production: *R*_*cl*_	*R*_*cl*_ = *W*_*cl*_*/P*_*cl*_	1.4%
Utilization ratio of solid-waste in cement production: *R*_*ce*_	*R*_*ce*_ = *W*_*ce*_*/P*_*ce*_	1.4%

## Data Records

There are seven excel files in our dataset. The seven excel involve the details of clinker rotary kilns, yearly production of the clinker and OPC cement products, fuel consumption and electricity consumption, total CO_2_ emissions of the OPC cement products, energy intensity indicators of the clinker and OPC cement products, utilization ratio of solid-waste in clinker and cement production, and CO_2_ emissions factors of the OPC cement products (Table 3). The entire database has been uploaded and publicly available at the *Figshare* repository^29^ and is available for download in excel format, and this dataset will be continuing to be updated annually.

**Table 3:** Overview of seven excel files in present dataset.

Number	Name	Details
1	clinker rotary kilns	Details of clinker rotary kilns for OPC cement plants in Guizhou of Southwest China 2019–2022
2	clinker and cement production	Yearly plant-level data of clinker and OPC cement production in Guizhou of Southwest China 2019–2022
3	fuel & electricity consumption	Yearly plant-level data of fuel consumption and electricity consumption in clinker and cement production in Guizhou of Southwest China 2019–2022
4	CO_2_ emissions	Yearly plant-level data of total CO_2_ emissions of OPC cement products in Guizhou of Southwest China 2019–2022
5	energy intensity	Yearly plant-level data of energy intensity indicators of clinker and OPC cement products in Guizhou of Southwest China 2019–2022
6	utilization ratio of solid-waste	Yearly plant-level data of utilization ratio of solid-waste in clinker and OPC cement production in Guizhou of Southwest China 2019–2022
7	CO_2_ emission factor	Yearly plant-level data of CO_2_ emission factor of OPC cement products in Guizhou of Southwest China 2019–2022

## Technical Validation

### Comparison of energy intensity with national standard

**Fig. 4:**
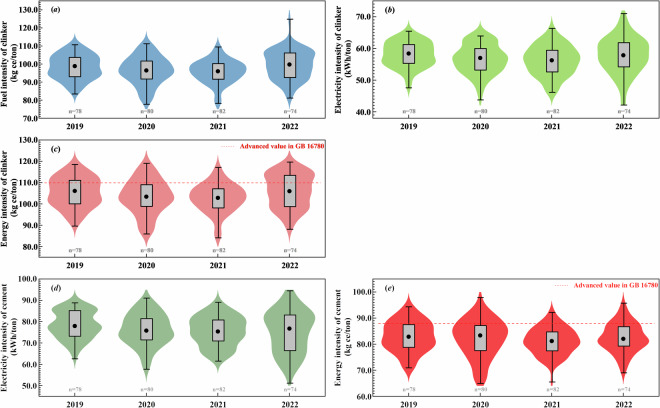
Comparison of energy intensity indicators: (**a**) fuel intensity of clinker, (**b**) electricity intensity of clinker, (**c**) energy intensity of clinker, (**d**) electricity intensity of cement, and (**e**) energy intensity of cement at plant-level in Guizhou from 2019 to 2022.

**Fig. 5:**
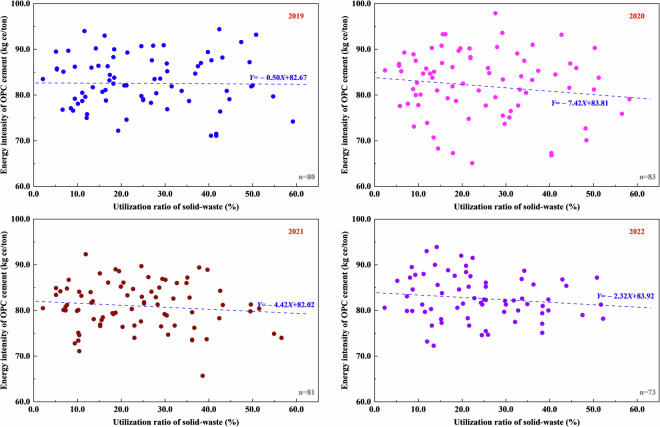
Benefit of utilization of solid-waste to energy intensity of cement at plant-level from 2019 to 2022.

We compared our energy intensity results with estimates in *The Norm of Energy Consumption per Unit Product of Cement* to validate the energy intensity data given in this dataset, which is firstly shown in Fig. 4. There is a decreasing trend in fuel intensity of clinker, electricity intensity of clinker, energy intensity of clinker, electricity intensity of cement, and energy intensity of cement from 2019 to 2022. The plant-level estimates for the energy intensity of cement in Guizhou lie in the middle range, while the annual average energy intensity indicators are better than the advanced-level values for that in the national standard of GB 16780. The utilization of solid-waste is also beneficial for the energy intensity of cement that demonstrated negative correlations with utilization ratio of solid-waste in cement production from 2019 to 2022, as shown in Fig. 5.

### Comparison of CO_2_ emissions factor with existing references

**Fig. 6:**
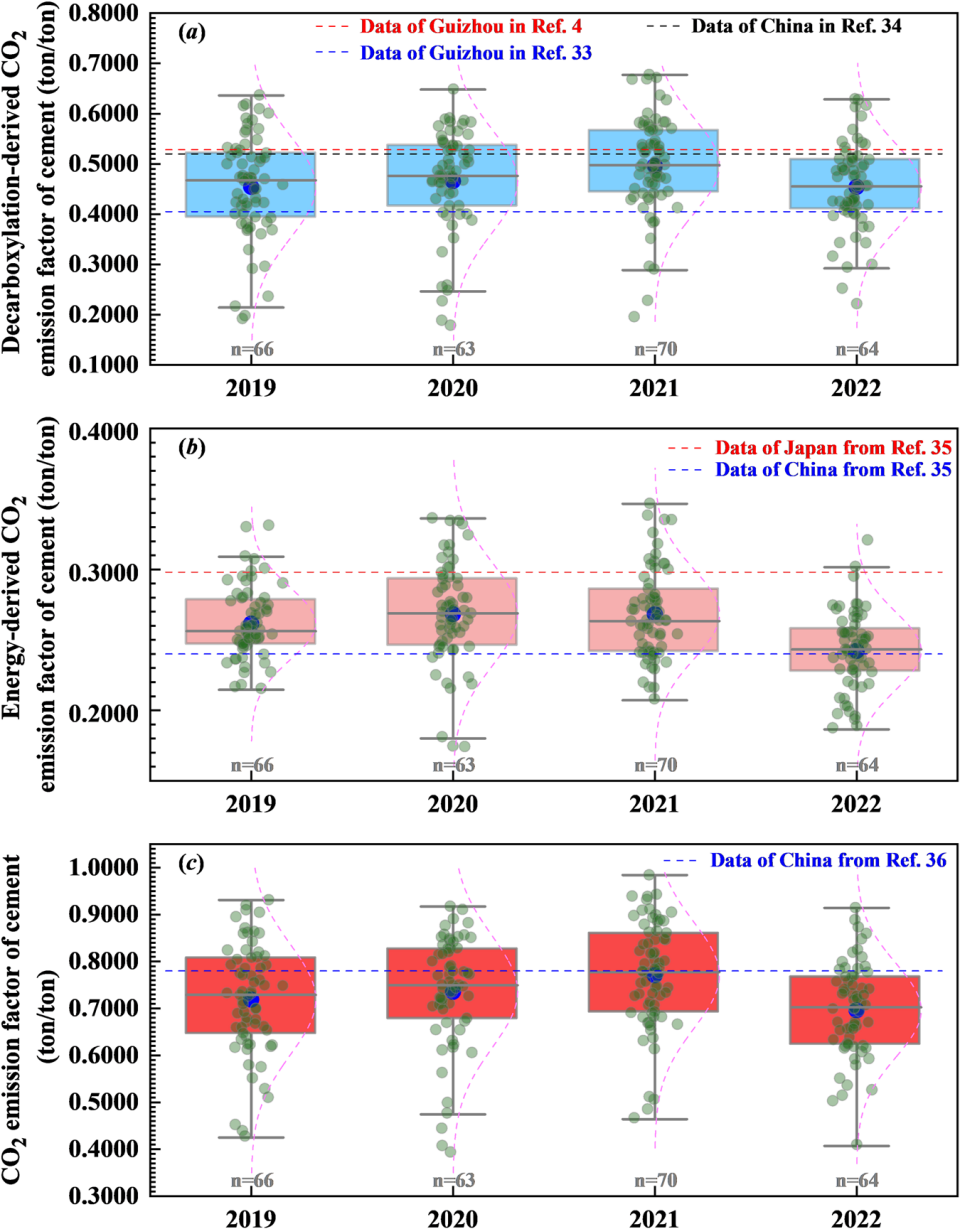
Comparison of (**a**) decarboxylation-derived CO_2_ emissions factor, (**b**) energy-derived CO_2_ emissions factor, and (**c**) CO_2_ emissions factor for cement at plant-level in Guizhou from 2019 to 2022.

Only a few references have provided provincial-level CO_2_ emissions factors for Guizhou’s cement industry. The decarboxylation-derived CO_2_ emissions factor of cement products in Guizhou is usually estimated to be 0.4050 ton CO_2_/ton^30^ or 0.5283 ton CO_2_/ton^4^, while that factor of the national level is estimated to be 0.5197 ton CO_2_/ton^31^. We plotted the plant-level CO_2_ emissions factors of cement in Guizhou from 2019 to 2022 in Fig. 6. We can find that the decarboxylation-derived CO_2_ emissions factor of cement products in this work is estimated to be (0.4567 ± 0.0641) ton CO_2_/ton in 2019, (0.4759 ± 0.0605) ton CO_2_/ton in 2020, (0.5046 ± 0.0612) ton CO_2_/ton in 2021, and (0.4589 ± 0.0482) ton CO_2_/ton in 2022, respectively. The results of decarboxylation-derived CO_2_ emission factor can reflect the differences in the cement manufacturing process and kilns at the plant-level.

The energy-derived CO_2_ emissions factor of cement products is closely related to its energy intensity. Since nearly half of the total capacity of clinker rotary kilns in Guizhou is provided by outdated equipment, the energy-derived CO_2_ emissions factor of cement products is higher than that of the national value^32^, which is been (0.2372 ± 0.0093) ton CO_2_/ton in 2019, (0.2694 ± 0.0235) ton CO_2_/ton in 2020, (0.2635 ± 0.0224) ton CO_2_/ton in 2021, and (0.2420 ± 0.0152) ton CO_2_/ton in 2022, respectively.

The CO_2_ emissions factor of cement in Guizhou, less than that of the national value^33^, is estimated to be (0.7263 ± 0.0808) ton CO_2_/ton in 2019, (0.7469 ± 0.0793) ton CO_2_/ton in 2020, (0.7444 ± 0.1153) ton CO_2_/ton in 2021, and (0.6944 ± 0.0712) ton CO_2_/ton in 2022, respectively.

### Limitations of Guizhou’s plant-level intensity of energy and CO_2_ emissions dataset

Although great efforts were made to guarantee the reliability of this dataset, the potential uncertainties in the data collection process were still unavoidable, mainly due to those specific time periods of the national industrial energy conservation inspections. Meanwhile, variations in fuel intensity and fuel-derived CO_2_ emissions are inevitable, which applying the annual average heat value of coal in various plants. And the impact of the transfer of clinker and cement between different plants or different cities are not considered in this work. These shortcomings should be considered by users.

## Usage Note

Although the dataset version in this manuscript relates to data collected from 2019 to 2022, the dataset of plant-level intensity of energy and CO_2_ emissions for Portland cement in Guizhou will be updated in every October or November, while the results of the national industrial energy conservation inspections released by DIIT of Guizhou. This dataset is openly accessible to the public.

This dataset is an important supplement and to existing energy intensity and CO_2_ emissions estimates at plant-level and provincial official emissions inventories that converges all regions of China. Provincial and county authorities can assess the impact of the production capacity and equipment in different cement plants on energy consumption and CO_2_ emissions by this dataset, which helps to implement the development of an effective carbon peaking policy and to realize the energy and environmental benefits. Managers and technicians can analyze this data to identify the superiority or inferiority in energy intensity and CO_2_ emissions and make informed decisions regarding energy consumption and waste utilization. This dataset is openly accessible to the public.

## Data Availability

There was no code used in the generation of the data in this work, an only Microsoft Excel is employed to process all the data.
